# Long‐distance effects of epidemics: Assessing the link between the 2014 West Africa Ebola outbreak and U.S. exports and employment

**DOI:** 10.1002/hec.3938

**Published:** 2019-08-28

**Authors:** Deliana Kostova, Cynthia H. Cassell, John T. Redd, Desmond E. Williams, Tushar Singh, Lise D. Martel, Rebecca E. Bunnell

**Affiliations:** ^1^ Division of Global Health Protection, Center for Global Health Centers for Disease Control and Prevention Atlanta Georgia

**Keywords:** epidemics, global health, health security, trade

## Abstract

Although the economic consequences of epidemic outbreaks to affected areas are often well documented, little is known about how these might carry over into the economies of unaffected regions. In the absence of direct pathogen transmission, global trade is one mechanism through which geographically distant epidemics could reverberate to unaffected countries. This study explores the link between global public health events and U.S. economic outcomes by evaluating the role of the 2014 West Africa Ebola outbreak in U.S. exports and exports‐supported U.S. jobs, 2005–2016. Estimates were obtained using difference‐in‐differences models where sub‐Saharan Africa countries were assigned to treatment and comparison groups based on their Ebola transmission status, with controls for observed and unobserved time‐variant factors that may independently influence trends in trade. Multiple model specification checks were performed to ensure analytic robustness. The year of peak transmission, 2014, was estimated to result in $1.08 billion relative reduction in U.S. merchandise exports to Ebola‐affected countries, whereas estimated losses in exports‐supported U.S. jobs exceeded 1,200 in 2014 and 11,000 in 2015. These findings suggest that remote disruptions in health security might play a role in U.S. economic indicators, demonstrating the interconnectedness between global health and aspects of the global economy and informing the relevance of health security efforts.

## 1 INTRODUCTION

Ebola virus disease, a rare but deadly hemorrhagic fever, has long captured the popular imagination as a broad symbol of a health threat that can defy containment. The disease is also a distinct and recurrent reality for some countries in Africa, where outbreaks have occurred for many years (Centers for Disease Control and Prevention [CDC], 2019). As of this writing, an ongoing epidemic has claimed close to 2,600 lives in Democratic Republic of the Congo (World Health Organization [WHO], 2019). The largest recorded outbreak, which emerged in 2014 and affected six countries in West Africa, caused 11,310 deaths over the course of 2 years and stirred global panic about the ability of contagious diseases to spread across borders (CDC, 2019).

The 2014 West Africa Ebola epidemic illustrated how inadequacies in local emergency preparedness could precipitate significant loss of life and capital. As Ebola infections spread to multiple countries in a matter of months, the outbreak revealed concerns that in an increasingly interconnected world, geographical distance no longer offers protection from emerging infectious threats. In August 2014, the outbreak was formally classified by the WHO as a public health emergency of international concern—“an extraordinary event … determined to constitute a public health risk to other States through the international spread of disease and to potentially require a coordinated international response.” (WHO, 2014, 2016). A coordinated response was initiated soon thereafter, when the United States joined other countries in endorsing the Global Health Security Agenda (GHSA) as a mechanism to improve health emergency preparedness globally (GHSA, 2017). GHSA focuses on strengthening core health security components such as disease surveillance, laboratory systems, health workforce development, and emergency management (CDC, 2017). Approximately half of the initial $1 billion contribution by the United States toward GHSA support was designated to Africa (Nuzzo, Cicero, & Inglesby, 2017). This commitment was renewed in 2017 with additional GHSA‐earmarked obligations of $454 million to the U.S. CDC and $245 million to the U.S. Agency for International Development (GHSA, 2018). U.S. investment in the global health security infrastructure aims to reinforce humanitarian and public health protections in resource‐limited environments, thereby strengthening domestic and international health safeguards.

GHSA investments take place in the context of a globally intertwined economy where links between health and economic outcomes both within and across countries can form a complex web of interactions. The role of health in economic growth has been the focus of extensive theoretical and empirical research (Acemoglu & Johnson, 2007; Barro, 2013; Bloom, Canning, & Sevilla, 2004; Ehrlich & Lui, 1991; World Bank, 2009). From the perspective of infectious diseases, epidemics can interfere with short‐term economic outcomes by changing expectations and deterring investment (Bloom & Canning, 2006), though their link to long‐term growth has not been established (Bloom & Canning, 2006; Bloom & Mahal, 1997). Nonetheless, the immediate economic costs of epidemics to affected regions can be considerable and are thus frequently assessed in the aftermath of notable outbreaks. For instance, the 2003 SARS outbreak was estimated to cost afflicted countries in the Western Pacific Region over $11 billion (Saywell, Fowler, & Crispin, 2003), with varying effects on national income growth and tourism (Beutels et al., 2009; Keogh‐Brown & Smith, 2008), whereas estimates of the regional economic burden imposed by the 2014 Ebola epidemic in West Africa range from from $2.8 billion in commerce and investment losses (World Bank, 2016) up to $53 billion in societal and economic cost (Huber, Finelli, & Stevens, 2018). Macroeconomic models, such as the G‐Cubed multicountry model previously used to assess the economic consequences of the SARS epidemic in Asia‐Pacific, have increased our understanding of the general equilibrium mechanisms that broaden the costs of epidemics beyond the cost of disease (Beutels, Edmunds, & Smith, 2008; Lee & McKibbin, 2004).

Although the consequences of epidemics to outbreak‐affected areas are often well documented, very little is known about if and how these might carry over into the economies of unaffected regions. Yet in a globalized world, such distal effects are likely. In the absence of direct pathogen transmission, global trade is one mechanism through which relatively remote epidemic outbreaks could reverberate to unaffected countries. Examining the impact of infectious outbreaks beyond the region of pathogen transmission offers an underexplored perspective of the interconnectedness between otherwise disparate countries and populations. This interconnectedness can have multiple angles; for instance, it has been observed that economic downturns in high‐income countries, such as the 2008 financial crisis in the United States, led to reductions in foreign health aid, altering the conditions for health development in lower income nations (Leach‐Kemon et al., 2012). Investigating the links between global health emergencies and U.S. economic outcomes can help with unfolding another layer in the complex relationship that describes the mutuality of global health and economic events.

This study explores the relevance of global health security priorities to U.S. economic outcomes by evaluating a major recent infectious disease outbreak, the 2014 West Africa Ebola epidemic, as a contributing factor to U.S. exports to countries in the affected region. We assess the role of the outbreak in two U.S. trade‐related outcomes: U.S. exports and exports‐supported U.S. jobs. The analysis employs a difference‐in‐differences (DD) framework, where sub‐Saharan Africa (SSA) countries were assigned to treatment and comparison groups based on their 2014 Ebola transmission status, including a primary treatment group of three countries with epidemic transmission (Guinea, Liberia, and Sierra Leone), a secondary treatment group of three countries that experienced limited Ebola cases (Mali, Nigeria, and Senegal), and a comparison group of 40 remaining SSA countries with no known cases. Adjusted differences in the trends of U.S. outcomes before, during, and after the 2014 Ebola period across treatment and comparison groups were used to assess Ebola‐attributable changes. These estimates represent potentially preventable losses in U.S. exports and employment from the spread of Ebola virus during the 2014 West Africa outbreak. In 2014 and 2015, the United States spent over $2.3 billion to support the Ebola response efforts in West Africa (CDC, 2016). The avertable economic cost of the epidemic to the United States may have been greater when domestic trade implications are considered. The findings demonstrate the interconnectedness of a world where even remote public health events cannot be considered in isolation, informing the relevance of global health security efforts.

## 2 DATA AND METHODS

### 2.1 Data

The outcomes examined in this study are U.S. merchandise exports to Ebola‐affected countries and U.S. jobs supported by these exports. Annual data on U.S. merchandise exports to countries in SSA from 2005 to 2016 were extracted from the Trade Policy Information System at the International Trade Administration, U.S. Department of Commerce (2018). Nominal values were converted into constant 2016 USD using consumer price index (CPI) deflation. Exports were defined as the value of all exported goods, excluding special provisions (North American Industry Classification System Code (NAICS) 99), which were subtracted because they can reflect donations or foreign aid. The value of services was not included because data on U.S. trade in services were not available for all SSA countries and years in the examined time period. In addition to total merchandise exports, detail on exports by merchandise type was obtained using NAICS two‐digit classification codes for agriculture and livestock products; oil, gas, minerals, and ores; manufacturing products; and other merchandise. Data on the annual number of U.S.‐based jobs supported by U.S. exports to SSA countries were obtained from Rasmussen and Xu (2016) for 2005–2015 and were not available for 2016.

### 2.2 Methods

The objective of this analysis is to evaluate the role of the 2014 West Africa Ebola outbreak in U.S. merchandise exports to Ebola‐affected countries and U.S. jobs supported by these exports. The analysis was performed using a DD framework, leveraging a natural experiment based on the presence of Ebola virus (strain specific to the 2014 West Africa outbreak) in some SSA countries and not others. In this framework, SSA countries were assigned to treatment and comparison groups according to their Ebola transmission status where “treatment” was defined as the presence of Ebola cases within a specified period of time. Because the intensity of the 2014 Ebola outbreak differed across countries affected by the virus, two treatment groups were defined: a primary treatment group composed of three countries that experienced epidemic transmission of Ebola virus: Guinea, Liberia, and Sierra Leone (Treatment Group 1), and a secondary treatment group composed of three countries that observed sporadic Ebola cases but no epidemic spread: Mali, Nigeria, and Senegal (Treatment Group 2). Forty remaining SSA countries with no known Ebola infections formed the comparison group (Angola, Benin, Botswana, Burkina Faso, Burundi, Cameroon, Cape Verde, Central African Republic, Chad, Comoros, Republic of Congo, Equatorial Guinea, Eritrea, Ethiopia, Gabon, Gambia, Ghana, Guinea‐Bissau, Ivory Coast, Kenya, Lesotho, Madagascar, Malawi, Mauritania, Mauritius, Mozambique, Namibia, Niger, South Africa, Rwanda, São Tomé and Príncipe, Seychelles, South Sudan, Sudan, Swaziland, Tanzania, Togo, Uganda, Zambia, and Zimbabwe). Two SSA countries were excluded from the comparison group: Somalia, because it does not have a trading relationship with the United States, and Democratic Republic of the Congo, because it experienced a concurrent but unlinked Ebola outbreak of a different strain unrelated to the main West Africa epidemic. All DD models controlled for macroeconomic factors that can independently influence trade fluctuations, including indicators of the strength of the U.S. dollar relative to other major world currencies, the relative domestic income of partner countries, and the relative price levels of partner countries. Country‐specific time trends were included to account for confounding from unobserved time‐variant factors unique to each country.

The model was specified as follows:

(1) $$Y_{\mathrm{jt}}=\beta_{0}+\beta_{1}\text{Treat}1_{j}+\beta_{2}\text{Treat}2_{j}+\beta_{3}2014+\beta_{4}\text{Post}2014+\beta_{5}\text{Treat}1_{j}*2014+\beta_{6}\text{Treat}1_{j}*\text{Post}2014+\beta_{7}\text{Treat}2_{j}*2014+\beta_{8}\text{Treat}2_{j}*\text{Post}2014+\beta_{9}X_{\mathrm{jt}}+\tau_{j}T+\varepsilon_{\mathrm{jt}},$$

where, in alternate specifications, *Y*_*jt*_ denotes the evaluated outcome for country *j* in year *t*; *Treat*1_*j*_ is a binary indicator equal to 1 if country *j* is one of the countries in Treatment Group 1 (Guinea, Liberia, and Sierra Leone) and zero otherwise; *Treat*2_*j*_ is a binary indicator equal to 1 if country *j* is one of the countries in Treatment Group 2 (Mali, Nigeria, and Senegal); 2014 is a binary indicator for year 2014, the peak year of transmission; Post2014 is a binary indicator for the period thereafter, which is 2015–2016 for exports models and 2015 for the jobs model; and *X*_*jt*_ is a vector of control variables that can influence trade indicators independent of Ebola considerations. These variables include the relative annual income and relative price level indicators for partner countries (country real gross domestic product [GDP] per capita relative to U.S. GDP and country CPI relative to U.S. CPI, respectively, sourced from International Monetary Fund, 2018, World Economic Outlook) and an indicator of the strength of the U.S. dollar relative to other major world currencies (the real trade‐weighted USD index, sourced from Federal Reserve Economic Data, 2018). We control for within‐country fluctuations in unobservable factors that might affect the examined outcomes by including country‐specific linear time trends (denoted by *τ*_*t*_*T*, the interaction terms between country dummy variables and a time trend) in order to reduce estimation bias from unknown and/or unmeasurable confounding factors within countries. The model in Equation (1) was estimated separately for each outcome variable using ordinary least squares with clustering of the standard errors by country and year. The estimators *β*_5_ and *β*_7_ represent the average treatment effect on the treated for each treatment group in 2014, whereas *β*_6_ and *β*_8_ represent the corresponding effects for the years following the start of the outbreak. Regression‐adjusted average differences between pre‐2014 and 2014 outcomes are represented by the sum of *β*_3_ and *β*_5_ for Treatment Group 1 and by the sum of *β*_3_ and *β*_7_ for Treatment Group 2.

### 2.3 Robustness checks

The validity of the DD methodology in estimating treatment effects is dependent on several conditions, including defining appropriate treatment and comparison groups and the assumption of parallel pretreatment trends in outcomes across groups. We employed several specification checks to evaluate the robustness of the analytic approach.

First, although the parallel trends assumption is not directly testable, we employed a common test of preexisting differences in outcomes trends. We limited the sample to observations before 2014 and regressed the outcome variable on binary indicators for each of the treatment groups, a linear time trend, and an interaction term between the two, in addition to all other control covariates. In this specification, a significant coefficient on the interaction term between treatment country status and time would be problematic as it would point to pretreatment trends in the outcomes of the treatment groups that are significantly different from those of the comparison group in violation of DD assumptions.

Second, we explored sensitivity of the results to changes in the composition of the comparison group. In a DD setting, selecting a comparison group that is similar to the treatment group(s) is important because higher group comparability supports the DD assumption that both groups would have experienced similar trends in outcomes in the absence of treatment. Although our main models control for a set of observed and unobserved differences across countries, we tested the robustness of the main results by selecting an alternative comparison group of countries that are in closer geographic proximity (and thus, presumably, more comparable in unobserved ways) to the treatment countries. This group consisted of all Ebola‐unaffected countries in the immediate West Africa subregion: Benin, Burkina Faso, Cape Verde, Gambia, Ghana, Guinea‐Bissau, Ivory Coast, Mauritania, Niger, São Tomé and Príncipe, and Togo. In this specification, finding treatment effects that are considerably different from those in the main models would be problematic as it might indicate use of inappropriate comparators at baseline.

Third, we further explored the assumption of comparability between the treatment and comparison groups by employing a model with propensity score weighting—an approach shown to improve the point estimate accuracy of average treatment effects in DD (Ryan, Burgess, & Dimick, 2015; Stuart et al., 2014). In this approach, treatment and comparison groups are balanced across a set of characteristics through the use of propensity score weights. To construct the weights, we estimated countries' probabilities of being in each of the three groups (Treatment Group 1, Treatment Group 2, and comparison group) using a logistic regression that predicted group assignment based on covariates that included per capita GDP, country population size, and consumer price level; weights were then equal to the inverse of the predicted probability of being in the group that the country belongs to (Lunceford & Davidian, 2004; Stuart et al., 2014). As in the previous sensitivity model, finding treatment effects that are considerably different from those in the main models would be problematic as it might indicate use of inappropriate comparators at baseline.

Fourth, we conducted a falsification test by estimating a model that substituted the original treatment groups with pseudotreatment groups of countries that did not have Ebola cases. The pseudotreatment groups were selected on the basis of their (unrealized) risk of Ebola transmission during the outbreak. At the time of the outbreak, three countries—Gambia, Guinea‐Bissau, and Ivory Coast—were identified as being highly susceptible to the spread of Ebola (Rainisch, Shankar, Wellman, Merlin, & Meltzer, 2015) but ultimately avoided hosting any infections. These countries formed the first pseudotreatment group. The second pseudotreatment group was composed of Benin, Burkina Faso, and Ghana—three proximal countries that remained unaffected while sharing borders with affected nations. Finding that the DD estimators in this model are similar to the DD estimators in the main models would be problematic as it would indicate that the main models reflect changes occurring throughout the region contemporaneously with but regardless of the spread of Ebola virus.

Fifth, a placebo test was performed by estimating a model that substituted the true timing of the 2014 outbreak with a false treatment indicator shifted to 3 years before the actual outbreak. In this model, finding significant DD estimators would be problematic as it would indicate that a divergence in exports trends between the treatment and comparison groups might have occurred well prior to the outbreak. To address the arbitrariness of selecting a specific year for the placebo treatment time, we used a permutation test, which has been shown to improve inference under certain conditions (Rokicki, Cohen, Fink, Salomon, & Landrum, 2018; Ryan et al., 2015). The permutation approach randomly reassigns outcome values within countries over time, thus randomizing the timing of treatment within countries; re‐estimates 50 iterations of the main model using the randomized outcome variable; and calculates new *p* values for the original DD estimator equal to the fraction of the iterations that produce estimators larger than those produced by the original model. This approach informs the robustness of the DD estimates to shifting the time of treatment without assigning a specific pseudotreatment year.

## 3 RESULTS

Table 1 describes the average annual value of exports by merchandise type across SSA countries for the periods before and after the outbreak. Overall, the value of U.S. exports to SSA averaged $397 million annually per country both before and after the outbreak (Table 1), exceeding $18 billion per year across all 46 countries in the region, largely from exports of manufacturing goods. Means of U.S. exports by group reveal group‐level differences in the before and after periods that hint at a post‐2014 divergence in exports across the treatment and comparison groups: on average, Ebola‐affected countries imported fewer goods from the United States in the outbreak period than before, whereas unaffected countries imported more (Table 1). In 2014–2016, countries in Treatment Group 1 received an average of $103 million in U.S. exports per country annually—down from an annual average of $106 million in 2005–2013. Treatment Group 2, a recipient of a much larger volume of exports from the United States than Treatment Group 1 due to a strong trading relationship with Nigeria, experienced a similar decline in exports from an annual average of $1,472 million per country in 2005–2013 to $1,332 million in 2014–2016. By contrast, the comparison group showed an increase in exports from an annual average of $337 million per country in 2005–2013 to $349 million in 2014–2016. Similar patterns were observed for the number of exports‐related U.S. jobs, whereas all groups showed declines in national income across the two periods (Table 1).

**Table 1:** Descriptive characteristics of treatment and comparison groups, annual country means

	2005–2013				2014–2016			
Characteristics	Treatment Group 1	Treatment Group 2	Comparison group	46 SSA countries	Treatment Group 1	Treatment Group 2	Comparison group	46 SSA countries
GDP per capita, constant 2016 USD	850	2,075	3,948	3,619	639	1,678	2,967	2,731
Value of U.S. merchandise exports, constant 2016 USD (millions)	106	1,472	337	397	103	1,332	349	397
Agriculture and livestock products (% of merchandise exports)	2.2	22.6	3.7	7.9	4.5	13.6	2.9	5.2
Oil, gas, minerals, and ores (% of merchandise exports)	0.1	0.2	0.5	0.5	2.2	0.9	1.6	1.5
Manufacturing products (% of merchandise exports)	83.3	71.9	87.0	83.7	78.9	74.7	91.6	87.7
Other (% of merchandise exports)	16.5	15.8	5.1	7.7	14.4	10.8	3.9	5.6
Number of U.S. jobs supported by exports	548	7,215	1,764	2,044	556	7,108	1,902	2,154

*Note*. Treatment Group 1 consists of three countries with epidemic transmission of 2014 Ebola: Guinea, Liberia, and Sierra Leone. Treatment Group 2 consists of three countries with limited cases of 2014 Ebola: Mali, Nigeria, and Senegal. The comparison group consists of 40 sub‐Saharan Africa countries without cases of 2014 Ebola.

Abbreviations: GDP, gross domestic product; SSA, sub‐Saharan Africa.

**Figure 1:**
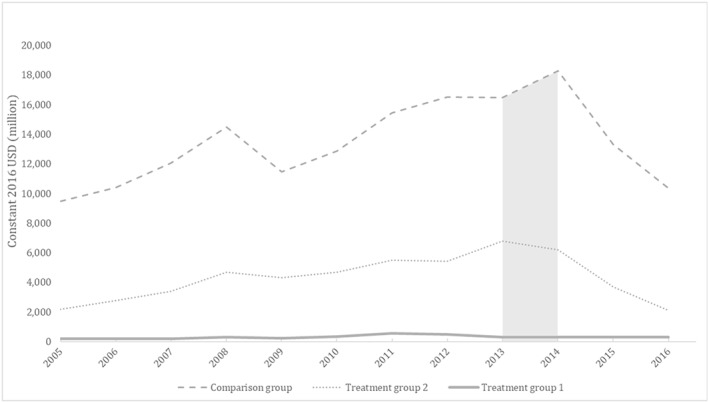
Aggregate U.S. merchandise exports, sub‐Saharan Africa countries, 2005–2016. Treatment Group 1 consists of three countries with epidemic transmission of 2014 Ebola: Guinea, Liberia, and Sierra Leone. Treatment Group 2 consists of three countries with limited cases of 2014 Ebola: Mali, Nigeria, and Senegal. The comparison group consists of 40 sub‐Saharan Africa countries without cases of 2014 Ebola. Shaded area denotes 2014, the year of peak Ebola virus transmission.

Trends in the aggregate value of U.S. exports by group are depicted in Figure 1. Until 2014, U.S. exports trends followed a similar pattern across groups, generally rising across SSA countries except for a collective dip during the 2009 U.S. recession. They diverged in 2014, the year of peak Ebola transmission, and declined thereafter. The 2014 contrast in U.S. exports trends across treatment and comparison groups motivates a DD approach to assessing the potential role of the Ebola outbreak in U.S. trade outcomes.

Estimates from the DD models in Equation (1) indicate that 2014 was significantly associated with a reduction in U.S. exports to Ebola‐affected countries relative to unaffected countries (Table 2). The relative annual decrease in U.S. exports in 2014 was estimated to be $90.4 million and $269.5 million per country for Treatment Groups 1 and 2, respectively, corresponding to an aggregate annual reduction of $1.08 billion for the year, mostly from a drop in manufacturing exports (Table 2). After 2014, the average treatment effects were no longer statistically significant for either treatment group, although a small bump of $4.6 million in agricultural exports was observed for Treatment Group 1. Year 2014 was estimated to bring a statistically significant decline in U.S.‐based jobs supported by exports to countries in Treatment Group 1, with an estimated average annual reduction of approximately 414 jobs per country for a total of 1,242 jobs (Table 2). For Treatment Group 2, the DD estimator was not statistically significant for 2014, but the post‐2014 DD estimator in the jobs model indicated a significant relative reduction of 3,681 jobs per country in year 2015, for a total of 11,043 U.S. jobs. Table 3 compares the unadjusted and regression‐adjusted average change in annual outcomes between the pre‐Ebola period and year 2014 for each group. Although the unadjusted changes were not statistically significant across groups, regression‐adjusted estimates indicated that a growth in outcomes in 2014 was observed for comparison countries only and was not accompanied by similar changes in the Ebola‐affected groups.

**Table 2:** Estimates from difference‐in‐differences models of U.S. merchandise exports to sub‐Saharan Africa countries

Variables of interest	U.S. merchandise exports					
	Total	Agricultural and livestock	Oil, gas, minerals, and ores	Manufacturing	Other	U.S. jobs
Treatment Group 1	−242.12* (134.59)	−52.41** (24.47)	0.47 (1.10)	−177.72 (110.61)	−12.50 (12.78)	−1480.02* (793.37)
Treatment Group 2	533.78 (467.98)	210.62 (164.59)	1.89 (1.63)	281.17 (259.39)	40.41 (39.47)	4092.17 (3376.03)
2014	90.93*** (24.61)	3.95 (4.29)	3.27** (1.46)	80.78*** (23.12)	3.10 (4.82)	344.85*** (107.97)
Post2014	73.08 (52.85)	25.71 (16.24)	2.41 (1.65)	21.56 (49.55)	23.85 (15.20)	224.86 (277.62)
Treatment Group 1 × 2014	−90.38*** (32.08)	3.24 (2.48)	−2.74* (1.54)	−85.40*** (31.83)	−5.23 (4.80)	−414.49*** (130.07)
Treatment Group 1 × Post2014	49.05 (54.48)	4.63** (2.35)	1.16 (3.24)	40.38 (54.73)	3.10 (13.59)	5.25 (162.34)
Treatment Group 2 × 2014	−269.50** (118.82)	−149.33 (127.66)	11.11 (9.15)	−5.34 (100.68)	−125.96 (110.04)	−1761.86 (1243.33)
Treatment Group 2 × Post2014	−1447.67 (1142.19)	−282.42 (212.64)	6.49 (4.28)	−854.50 (682.39)	−317.22 (256.49)	−3681.28* (2179.28)
U.S. currency value index	−9.28*** (2.66)	−1.18* (0.67)	−0.07 (0.09)	−5.95** (2.39)	−2.11^***^ (0.63)	−19.61 (15.55)
Partner country GDP per capita relative to U.S.	1643.81 (1349.34)	43.81 (50.16)	4.01 (3.65)	1573.99 (1287.51)	23.21 (36.84)	9829.41 (7681.30)
Partner country CPI relative to U.S.	−434.44 (290.88)	−128.19* (71.23)	2.31 (2.22)	−272.61 (206.99)	−35.57 (40.63)	−2612.28* (1582.24)
*N* (country‐years)	546	546	546	546	546	500

*Note*. Treatment Group 1 consists of three countries with epidemic transmission of 2014 Ebola: Guinea, Liberia, and Sierra Leone. Treatment Group 2 consists of three countries with limited cases of 2014 Ebola: Mali, Nigeria, and Senegal. The comparison group consists of 40 sub‐Saharan Africa countries without cases of 2014 Ebola. Estimates obtained from linear models with clustering by country and year. All models include controls for country‐specific time trends (not shown). Standard errors in parentheses. Models of U.S. exports are based on data for 2005–2016. Model of U.S. jobs is based on data for 2005–2015. U.S. exports in constant 2016 USD (millions).

* Statistically significant at 10% level.

** Statistically significant at 5% level.

*** Statistically significant at 1% level.

Abbreviations: CPI, consumer price index; GDP, gross domestic product.

**Table 3:** Unadjusted and regression‐adjusted average annual differences in U.S. exports and U.S. exports‐supported jobs between 2005–2013 and 2014, by group

Variables of interest	2005–2013	2014	Unadjusted difference (%)	Regression‐adjusted difference (%)
U.S. exports (million constant 2016 USD)				
Treatment Group 1	105.7	103.4	−2%	.5%
Treatment Group 2	1472.3	2067.2	40%	12%
Comparison group	337.3	456.7	35%	27%***
U.S. jobs (number)				
Treatment Group 1	548.3	520.0	−5%	−13%
Treatment Group 2	7215.4	8052.3	12%	−20%
Comparison group	1764.1	2141.8	21%	20%***

*Note*. Treatment Group 1 consists of three countries with epidemic transmission of 2014 Ebola: Guinea, Liberia, and Sierra Leone. Treatment Group 2 consists of three countries with limited cases of 2014 Ebola: Mali, Nigeria, and Senegal. The comparison group consists of 40 sub‐Saharan Africa countries without cases of 2014 Ebola.

* Statistically significant at 10% level.

** Statistically significant at 5% level.

*** Statistically significant at 1% level.

### 3.1 Robustness checks

The model used to test for differential exports trends across groups did not find evidence of significant trend differences predating the outbreak, evidenced by the insignificant interaction terms between treatment group and time (Table 4). This finding is supportive of the main model assumption that the effects represented by the DD estimators in Equation (1) are not a reflection of preexisting differentials and may thus be plausibly linked to the 2014 introduction of the virus.

**Table 4:** Summary of estimates from model of pre‐Ebola trends in U.S. exports, 2005–2013

Variables of interest	Coefficient
Treatment Group 1	−364.56** (164.96)
Treatment Group 2	563.73*** (124.86)
Time trend	36.53 (28.57)
Treatment Group 1 × Time Trend	16.32 (23.17)
Treatment Group 2 × Time Trend	143.34 (191.73)
*N*	408

*Note*. Treatment Group 1 consists of three countries with epidemic transmission of 2014 Ebola: Guinea, Liberia, and Sierra Leone. Treatment Group 2 consists of three countries with limited cases of 2014 Ebola: Mali, Nigeria, and Senegal. The comparison group consists of 40 sub‐Saharan Africa countries without cases of 2014 Ebola. Estimates obtained from linear models with clustering by country and year. All models include controls for country‐specific time trends, U.S. currency value index, partner country gross domestic product per capita relative to U.S., and partner country consumer price index relative to U.S. (not shown). U.S. exports in constant 2016 USD (millions). Standard errors in parentheses.

* Statistically significant at 10% level.

** Statistically significant at 5% level.

*** Statistically significant at 1% level.

After narrowing down the original comparison group of 40 SSA countries to a smaller group of 11 countries with greater proximity to the outbreak area—unaffected countries located in the West Africa subregion—the DD estimators remained similar to those obtained from the main models (Table 5, Specification 2). On aggregate, the 2014 effect on U.S. exports to the six treatment countries was nearly identical in magnitude to the corresponding estimate from the main models ($1.07 billion vs. $1.08 billion). Our next robustness check, which applied propensity score weighting to the main specification, resulted in lower aggregate effects ($754 million) due to a decrease in the DD estimator for Treatment Group 2 in the propensity score model compared with the main model (Table 5, Specification 3). Because Treatment Group 2 contains Nigeria, a country with a considerably larger economy and trade volume relative to its neighbors in the region, the use of propensity score weighting could have corrected down the disproportionate influence of Nigeria in driving the main model effects for Treatment Group 2.

**Table 5:** Summary of estimates from robustness models

Variables of interest	Main model	Sensitivity model: limit comparison countries to West Africa only	Sensitivity model: propensity score weighting of country groups	Falsification model: pseudotreatment groups	Placebo model: pseudooutbreak year	Permutation test: randomized treatment time
	(1)	(2)	(3)	(4)	(5)	(6)
Treatment Group 1 × 2014	−90.38*** (0.00)	−119.83* (0.09)	−107.89* (0.09)	−30.87 (0.46)	45.53 (0.16)	−90.38* (0.08)
Treatment Group 1 × Post‐2014	49.05 (0.37)	−8.99 (0.92)	61.28 (0.38)	144.66** (0.04)	−48.03 (0.31)	49.05 (0.52)
Treatment Group 2 × 2014	−269.50** (0.02)	−235.86* (0.06)	−143.57* (0.09)	−107.75 (0.18)	574.55 (0.16)	−269.50 (0.74)
Treatment Group 2 × Post‐2014	−1447.67 (0.20)	−1290.18 (0.28)	−168.82 (0.61)	−256.57 (0.12)	343.88 (0.29)	−1447.67** (0.02)
*N*	546	204	546	474	546	546

*Note*. (1) Main model. Treatment Group 1 consists of three countries with epidemic transmission of 2014 Ebola: Guinea, Liberia, and Sierra Leone. Treatment Group 2 consists of three countries with limited cases of 2014 Ebola: Mali, Nigeria, and Senegal. The comparison group consists of 40 sub‐Saharan Africa countries without Ebola cases. (2) Sensitivity model: Treatment groups are the same as in main model (1); comparison group is limited to 11 unaffected countries in the same SSA subregion as Ebola‐affected countries (Benin, Burkina Faso, Cape Verde, Gambia, Ghana, Guinea‐Bissau, Ivory Coast, Mauritania, Niger, São Tomé and Príncipe, and Togo). (3) Sensitivity model: Treatment and comparison countries are the same as in main model (1); observations in each group are weighted using propensity scores. (4) Falsification model: Pseudotreatment Group 1 consists of three countries identified as being at high risk for 2014 Ebola transmission but experiencing no Ebola cases (Gambia, Guinea‐Bissau, and Ivory Coast). Pseudotreatment Group 2 consists of three countries in geographic proximity to Ebola‐affected countries but experiencing no Ebola cases (Benin, Burkina Faso, and Ghana). The comparison group consists of 34 remaining sub‐Saharan Africa countries without Ebola cases. (5) Placebo model: Treatment and comparison countries are same as in main model (1); pseudooutbreak year set to 2011. (6) Permutation test: Treatment and comparison countries are the same as in main model (1); outcome values are randomized within countries over multiple iterations without specifying a single pseudotreatment year. All estimates obtained from linear models with clustering by country and year. All models include controls for country‐specific time trends, U.S. currency value index, partner country gross domestic product per capita relative to U.S., and partner consumer price index relative to U.S. (not shown). U.S. exports in constant 2016 USD (millions). *p* values in parentheses.

* Statistically significant at 10% level.

** Statistically significant at 5% level.

*** Statistically significant at 1% level.

A falsification test using pseudotreatment groups of countries that were at an elevated risk for 2014 Ebola due to their proximity but had no actual infections yielded no evidence of Ebola‐attributed reduction in U.S. exports to those countries (Table 5, Specification 4). The 2014 DD estimators in this model were not statistically significant, which supports the assumption that the 2014 treatment effects estimated by the main models were not an expression of shared regional trade fluctuations coincidental to the outbreak. Interestingly, a positive interaction term between Pseudotreatment Group 1 (Gambia, Guinea‐Bissau, and Ivory Coast) and the post‐2014 period indicated a relative increase in U.S. exports to these countries in 2015–2016—a possible sign of a compensatory shift in exports from the affected countries to some of their neighbors.

In addition to exploring the validity of the treatment and comparison groups, we conducted checks on the DD estimators from the perspective of treatment timing. A placebo model that substituted the original treatment indicator, 2014, with a false outbreak year, 2011, revealed no significant treatment effects (Table 5, Specification 5), supporting the assumption that the main model treatment effects were not an expression of spurious divergence in exports trends predating the outbreak. Addressing concerns about the arbitrary choice of a false outbreak year in the preceding placebo model, we used a permutation test that randomly shifts the timing of treatment within countries, reproducing the value of the DD estimator over multiple iterations. This test confirmed the statistical significance of the main model estimator for Treatment Group 1 (Table 5, Specification 6). For Treatment Group 2, the significance of the DD estimator shifted from 2014 to post‐2014. Because the post‐2014 treatment effect for Treatment Group 2 ($1,447.7 million) is larger in magnitude than the 2014 effect for this group ($269.5 million), this finding implies a possibility that the aggregate Ebola‐attributed reduction in U.S. exports might be more extensive than estimated in the main models.

## 4 DISCUSSION

This study suggests that U.S. exports and U.S. exports‐supported jobs were adversely affected by the 2014 outbreak of Ebola virus disease in West Africa. DD estimates indicate that the year of peak transmission, 2014, resulted in $1.08 billion aggregate reduction in U.S. merchandise exports to Ebola‐affected countries relative to unaffected countries in the region. Estimated losses in U.S. jobs supported by exports in Ebola‐affected countries exceeded 1,200 in 2014 and 11,000 in 2015.

Because the countries affected by the 2014 West Africa Ebola outbreak are relatively minor trading partners for the United States, it is plausible that epidemics affecting larger partner countries would be more consequential. For example, the East Asia and Pacific region is one area with key U.S. trade relationships that has an elevated risk of emerging epidemics, where Indonesia alone is both a top destination for U.S. exports (Office of the U.S. Trade Representative, 2018) and an identified hotspot for potential infectious outbreaks (Perkins, Patrick, Patel, & Fenwick, 2007) and where hypothetical outbreak scenarios have suggested that billions in U.S. exports could be at stake in the event of an infectious disease epidemic (Bambery et al., 2018). The expected worldwide economic loss from a major influenza pandemic has been estimated at 0.6% of global income (Fan, Jamison, & Summers, 2017), with country‐specific pandemic simulations predicting losses of up to 6% of GDP for several Western European countries (Keogh‐Brown, Smith, Edmunds, & Beutels, 2010) and up to 7.3% of GDP for Australia (Verikios, McCaw, McVernon, & Harris, 2012). Economic losses from modeled influenza pandemics are projected to be higher for countries with stronger trade ties to other countries (Verikios, Sullivan, Stojanovski, Giesecke, & Woo, 2011). Currently, the United States has strong trade links to 49 countries identified as GHS priority countries by the CDC. In 2015, these 49 countries accounted for over USD 308 billion in U.S. exports, supporting over 1.6 million U.S. export‐related jobs (Cassell et al., 2017). Minimizing health security threats in these countries may help support strategies for a positive trade balance in the United States.

Although health security protections can have global economic benefits, their primary goal is preventing the loss of life through strengthening capacity for disease containment. Since the establishment of GHSA, some countries in Africa have observed expanded laboratory capabilities for detecting pathogens, strengthened field networks of public health professionals, and shorter time for detection of epidemic outbreaks (CDC, 2017). In some countries, such as in Sierra Leone, advances in surveillance have been accompanied by improved vaccination rates for children (CDC, 2017). Continued commitment to GHSA priorities could thus help reinforce broad advancements in public health alongside outbreak control in vulnerable areas.

While this study does not shed light on the actual mechanisms through which epidemics can interfere with trade, those can be many‐sided. For example, epidemics could disrupt travel and transportation logistics through curfews, travel restrictions, and market closures and could lower foreign direct investment, shift the internal resource allocations of affected countries, and affect currency values. Economic relationships are particularly sensitive to the uncertainty and alarm that can be generated by public health emergencies: the economic disturbance from negative expectations can disproportionately exceed the actual number of infections (Bloom & Canning, 2006). Changes in economic behavior driven by risk perceptions of individuals and industry can have wide‐ranging repercussions in a macroeconomic context (Beutels et al., 2008; Lee & McKibbin, 2004; McKibbin & Stoeckel, 2018).

The main models of this study, as well as most robustness checks, imply that the estimated effect of the West Africa Ebola outbreak was statistically significant for 2014 only—the year of peak transmission. However, the statistical absence of post‐2014 effects on exports to Ebola‐affected countries may be less likely to represent a post‐Ebola rebound in exports than a reflection of a shared decline in U.S. exports across all of SSA. Although Ebola did not fully subside until 2016, both affected and unaffected countries experienced a downturn in U.S. exports after 2014 (Figure 1), possibly marking the spread of subsequent economic repercussions throughout the entire region. The shared post‐2014 decline in U.S. exports across all groups in SSA limits the ability of the present DD method to derive post‐2014 treatment effects from differences across groups. However, one of the robustness models in this study, a permutation test, suggests that significant post‐2014 effects may be present for Treatment Group 2. Expanding the analysis with more years of data may allow more precise identification of long‐term effects.

The analysis has several other limitations. It is restricted to merchandise exports because data on the value of service exports were not available for all countries in the region. It evaluates trade disruptions associated only with the presence of actual infections in a country—that is, it does not evaluate secondary effects resulting from the increased risk of infection and rising negative expectations in SSA countries unaffected by actual contagion. Such secondary effects are likely to have a wider geographic scope: previous evaluation of African economies has shown that SSA countries at a geographic distance from the outbreak experienced considerable post‐Ebola economic decline (World Bank, 2016). In the present analysis, secondary Ebola effects on U.S. exports—those specific to SSA countries that did not have Ebola infections—are not estimated given the lack of an obvious comparison group in this scenario. Nonetheless, it is likely that if all SSA economies suffered following the West Africa outbreak, so would have their ability to serve as destinations for U.S. exports.

The study has several strengths. To our knowledge, it is the first to establish a link between a distal public health emergency and select U.S. economic indicators. The analytic framework compares trends in U.S. exports to SSA countries with different levels of Ebola virus transmission before and after the 2014 emergence of the outbreak while controlling for unobserved time‐varying country characteristics that might independently determine trade outcomes. A number of specification checks provide empirical support for the validity of the analytic approach, helping to reinforce treatment effects.

Alongside international partners, the United States has supported the goals of the Global Health Security Agenda in strengthening country readiness for containing infectious disease outbreaks. The three main elements of GHS are the prevention of outbreaks, early detection of threats, and capacity for rapid response. Ongoing development and maintenance of the infrastructure behind these elements is essential but not guaranteed and depends on continued support from the United States and other high‐resource countries. Our findings illustrate the associations between health and economic events that connect countries notwithstanding of geographical distance, informing the relevance of global health security efforts.
